# Sex-Specific Transmission of Anxiety Disorders From Parents to Offspring

**DOI:** 10.1001/jamanetworkopen.2022.20919

**Published:** 2022-07-12

**Authors:** Barbara Pavlova, Alexa Bagnell, Jill Cumby, Emily Howes Vallis, Sabina Abidi, David Lovas, Lukas Propper, Martin Alda, Rudolf Uher

**Affiliations:** 1 Department of Psychiatry, Dalhousie University, Halifax, Nova Scotia, Canada; 2 Nova Scotia Health, Halifax, Nova Scotia, Canada; 3 IWK Health Centre, Halifax, Nova Scotia, Canada

## Abstract

**Importance:**

Although anxiety disorders are known to run in families, the relative contribution of genes and environment is unclear. Patterns of sex-specific transmission of anxiety may point to different pathways in how parents pass anxiety disorders down to their children; however, the association of parent and offspring sex with the transmission of anxiety disorders has not been previously studied.

**Objective:**

To examine whether the transmission of anxiety from parents to children is sex specific.

**Design, Setting, and Participants:**

This cross-sectional family study recruited participants from the general population (enriched for familial risk of mood disorders) in Nova Scotia, Canada, from February 1, 2013, to January 31, 2020.

**Exposures:**

Anxiety disorder in the same-sex or opposite-sex parent.

**Main Outcomes and Measures:**

Semistructured interviews were used to establish lifetime diagnoses of anxiety disorder in parents and offspring. The association between anxiety disorder in the same-sex or opposite-sex parent and anxiety disorders in the offspring was tested with logistic regression.

**Results:**

A total of 398 offspring (203 female offspring with a mean [SD] age of 11.1 [3.7] years and 195 male offspring with a mean [SD] age of 10.6 [3.1] years) of 221 mothers and 237 fathers participated in the study. Anxiety disorders in the same-sex parent (odds ratio [OR], 2.85; 95% CI, 1.52-5.34; *P* = .001) were associated with increased rates of anxiety disorders in the offspring, whereas anxiety disorders in the opposite-sex parent (OR, 1.51; 95% CI, 0.81-2.81; *P* = .20) were not. Sharing a household with a same-sex parent without anxiety was associated with lower rates of offspring anxiety (OR, 0.38; 95% CI, 0.22-0.67; *P* = .001), but the presence of an opposite-sex parent without anxiety was not (OR, 0.96; 95% CI, 0.56-1.63; *P* = .88).

**Conclusions and Relevance:**

In this cross-sectional study of families, an association between the same-sex parent’s anxiety disorder and anxiety disorders in offspring suggests an environmental mechanism, such as modeling. Future studies should establish whether treating parents’ anxiety may protect their children from developing an anxiety disorder.

## Key Points

**Question:**

Does the likelihood of transmission of anxiety disorders from parents to children differ between same-sex and opposite-sex parent-offspring pairs?

**Findings:**

In this cross-sectional study of 398 offspring from the general population in Canada (enriched for familial risk of mood disorders), those with a same-sex parent with an anxiety disorder were more likely to have an anxiety disorder than offspring with an opposite-sex parent with an anxiety disorder.

**Meaning:**

This finding suggests a possible role of environmental factors, such as modeling and vicarious learning, in the transmission of anxiety from parents to children.

## Teaser

This cross-sectional study of the general population in Nova Scotia, Canada, examines the association of parent and offspring sex with the transmission of anxiety disorders.

## Introduction

Anxiety disorders are the most common psychiatric disorders.^1,2^ They often start early in life^2^ and are prospectively associated with depression, bipolar disorder, educational underachievement, harmful substance use, and suicide.^3,4,5,6,7^ One of the strongest known risk factors for developing an anxiety disorder is having a parent with an anxiety disorder,^8,9^ an effect that increases with 2 parents being affected.^10,11,12^ This association could be attributable to the parents passing on genetic risk to their offspring and the impact parents have on their children’s environment. Although a number of genetic variants associated with anxiety disorders have been identified,^13,14^ some studies^15,16^ have concluded that the association of environment with the transmission of anxiety from parents to children is greater than that of the genetics.

Because children share a similar number of autosomal genetic variants with their mothers and their fathers, patterns of sex-specific transmission of psychiatric disorders from parents to offspring may point to different underlying mechanisms. In psychosis, for example, the stronger contribution of the opposite-sex parent to the transmission suggests a role of the X chromosome.^17,18,19^ On the other hand, if the contribution of the same-sex parent with a disorder is greater, it may suggest that modeling and vicarious learning from the same-sex parent play a role in the transmission. The role of modeling in anxiety transmission from parents to offspring has been highlighted before in experimental studies.^20,21^

To our knowledge, the role of parent and offspring sex in the transmission of anxiety disorders has not been previously studied. Many studies recruited only mothers or struggled to recruit enough fathers. In addition, not all parents reside with their children, and those who do not are likely exerting smaller influence on their offspring. In the current study, we explore the association of parent and offspring sex with the transmission of anxiety disorders using a sample of mothers, fathers, and their offspring while taking into account whether the parent and offspring share the same household.

## Methods

### Participants

Participants were recruited as a part of the Families Overcoming Risks and Building Opportunities for Well-being (FORBOW) project^22^ from February 1, 2013, to January 31, 2020, in Nova Scotia, Canada. The sample is enriched for offspring of parents with mood disorders. Families with a parent with a mood disorder were recruited through the parents’ clinicians. Control families were recruited from the same schools and neighborhoods as the affected families. We included families with 1 or more offspring between the ages of 5 and 21 years with an IQ greater than 70. The study was approved by the research ethics board of Nova Scotia Health. Participants provided written informed consent. Children who did not have the capacity to make an informed decision gave assent, and their legal guardian provided written informed consent on their behalf. We followed the Strengthening the Reporting of Observational Studies in Epidemiology (STROBE) reporting guideline^23^ for observational cross-sectional studies.

### Measurement

Parents and offspring were assessed by different assessors. We always attempted to assess both biological parents. We assessed parents’ *Diagnostic and Statistical Manual of Mental Disorders* (Fourth Edition) (*DSM-IV*) and *Diagnostic and Statistical Manual of Mental Disorders* (Fifth Edition) (*DSM-V*) psychiatric diagnoses using the Schedule for Affective Disorders and Schizophrenia^24^ and the Structured Clinical Interview for *DSM-5* Disorders (SCID-5).^25^ Most interviews were completed face to face. In a few cases, interviews were conducted over the telephone. We confirmed all parental diagnoses in consensus meetings with psychiatrists unaware of offspring psychopathology. We established offspring *DSM-IV* and *DSM-5* diagnoses using the Kiddie Schedule for Affective Disorders and Schizophrenia^26^ and SCID-5.^25^ We finalized all offspring diagnoses in consensus meetings with a child and adolescent psychiatrist. The assessors and the psychiatrists were blind to the parents’ diagnoses. We assessed offspring general cognitive ability with the Wechsler Abbreviated Scale of Intelligence, Second Edition.^27^ We also collected data on who shared the household with the offspring. The data on participants' race and ethnicity were based on self-report or parent report.

### Statistical Analysis

The outcome variable was the diagnosis of an anxiety disorder in the offspring, defined as a diagnosis of 1 or more of generalized anxiety disorder, social anxiety disorder, separation anxiety disorder, panic disorder, agoraphobia, simple phobia, anxiety disorder not otherwise specified, obsessive compulsive disorder, or posttraumatic stress disorder. The independent variables included parental diagnoses of anxiety disorders. We also stratified the analysis by the sex of the parent and the sex of the offspring.

We tested the associations between parent diagnoses and offspring diagnoses in logistic regression models with robust SEs to provide estimates that accounted for clustering of individuals within families. We included offspring age and sex as covariates in all analyses. We first tested the association between parental anxiety disorders and anxiety disorders in offspring. To control for mood disorders, we tested the association between parental diagnoses of mood disorders and the anxiety disorder diagnoses in offspring. We also tested the relative associations of parental mood disorders and anxiety disorders with the anxiety disorders in offspring in a logistic regression model that included parental mood disorder and parental anxiety disorder as independent variables.

We examined the sex-specific parent-of-origin hypothesis as the association of an anxiety disorder in the same-sex and opposite-sex parent with the anxiety disorder in offspring. Furthermore, we separately tested the associations of paternal and maternal anxiety disorders with anxiety disorders in sons and daughters. Finally, to distinguish between the absence of a diagnosis in a parent and the absence of a parent from a household, we tested the association of sharing the household with a parent without anxiety disorder with the presence of an anxiety disorder in offspring, separately for same-sex and opposite-sex parents. Tests were 2-sided, and *P* < .05 was considered statistically significant. Statistical analyses were performed with Stata software, version 17.0 (StataCorp LLC).^28^

## Results

### Participants

The participants were 398 offspring (203 female offspring with a mean [SD] age of 11.1 [3.7] years and 195 male offspring with a mean [SD] age of 10.6 [3.1] years; 352 [88.4%] White and 46 [11.6%] other [African, Chinese, East Indian, Inuit, Mi’kmaq, Métis, or multiracial]) of 221 mothers and 237 fathers. Offspring characteristics are given in Table 1.

**Table 1.** Offspring Characteristics by Parent Diagnosis

Parental diagnosis of mood or psychotic disorder	No. of offspring	Offspring age, mean (SD), y	Offspring anxiety disorder, No. (%)
No diagnosis			
Male offspring	48	10.7 (2.5)	13 (27.1)
Female offspring	48	10.9 (3.2)	10 (20.8)
Depression			
Male offspring	89	10.4 (3.3)	18 (20.2)
Female offspring	92	11.3 (3.8)	33 (35.9)
Bipolar disorder			
Male offspring	44	10.7 (3.7)	15 (34.1)
Female offspring	45	10.9 (4.2)	17 (37.8)
Schizophrenia			
Male offspring	14	10.3 (2.4)	1 (7.1)
Female offspring	18	10.8 (3.5)	1 (5.6)

Both biological parents of 231 of the 251 offspring (92.0%) who lived with both biological parents completed diagnostic interviews about their own psychiatric diagnoses. Both biological parents of 68 of the 147 offspring (46.3%) who did not share a household with both biological parents also completed a diagnostic interview focusing on their own psychiatric diagnoses.

A total of 221 mothers were assessed at a mean (SD) age of 39.8 (6.2) years. Of the assessed mothers, 93 (42.1%) had a diagnosis of major depressive disorder, 45 (20.4%) had a diagnosis of bipolar disorder, and 11 (5.0%) had a diagnosis of schizophrenia. A total of 101 mothers (45.7%) had a diagnosis of 1 or more anxiety disorders, most of them comorbid with major mood disorders (Table 2).

**Table 2.** Characteristics of Mothers and Fathers

Parental diagnosis of mood or psychotic disorder	Mothers, No./total No. (%)		Fathers, No./total No. (%)	
	Anxiety disorder	No anxiety disorder	Anxiety disorder	No anxiety disorder
No mood or psychotic disorder	7/72 (9.7)	65/72 (90.3)	8/106 (7.5)	98/106 (92.5)
Schizophrenia	2/11 (18.2)	9/11 (81.8)	1/7 (14.3)	6/7 (85.7)
Bipolar disorder	32/45 (71.1)	13/45 (28.9)	11/17 (64.7)	6/17 (35.3)
Major depressive disorder	60/93 (64.5)	33/93 (35.5)	20/42 (47.6)	22/42 (52.4)
Total	101/221 (45.7)	120/221 (54.3)	40/172 (23.3)	132/172 (76.7)

A total of 172 fathers were assessed at a mean (SD) age of 42.0 (7.1) years. Of the assessed fathers, 42 (24.4%) had a diagnosis of major depressive disorder, 17 (9.9%) had a diagnosis of bipolar disorder, and 7 (4.1%) had a diagnosis of schizophrenia. Forty assessed fathers (23.3%) had a diagnosis of 1 or more anxiety disorders, most of them comorbid with major mood disorders (Table 2).

Analyses that required diagnostic information on both parents were conducted in a reduced sample of 299 offspring (149 sons and 150 daughters) who had both biological parents assessed. This reduced sample was similar in age and sex distribution to the whole sample (150 [50.2%] female; mean [SD] age, 10.7 [3.4] years).

### Anxiety Disorders in Offspring

Of the 398 offspring, 108 (27.1%) received a diagnosis of 1 or more lifetime anxiety disorders. Common anxiety disorder diagnoses included generalized anxiety disorder (31 offspring [7.8%]), social anxiety disorder (25 offspring [6.3%]), separation anxiety disorder (34 offspring [8.6%]), specific phobia (32 offspring [8%]), and anxiety disorder not otherwise specified (20 offspring [5%]). Eleven offspring (2.8%) fulfilled criteria for obsessive compulsive disorder. Panic disorder, agoraphobia, and posttraumatic stress disorder were uncommon, with fewer than 6 offspring meeting diagnostic criteria for each. Rates of anxiety disorders increased with offspring age, from 21 of 149 (14.1%) in those younger than 9 years to 29 of 56 (51.8%) in those older than 15 years. Anxiety disorders were similarly common among male (47 of 195 [24.1%]) and female (61 out of 203 [30.1%]) offspring. Anxiety disorders were more common in offspring with missing diagnostic information on 1 biological parent (36 of 99 [36.4%]) than offspring with diagnostic information on both parents (72 of 299 [24.1%]).

### Parent Anxiety Disorders and Anxiety Disorders in Offspring

**Figure 1.**
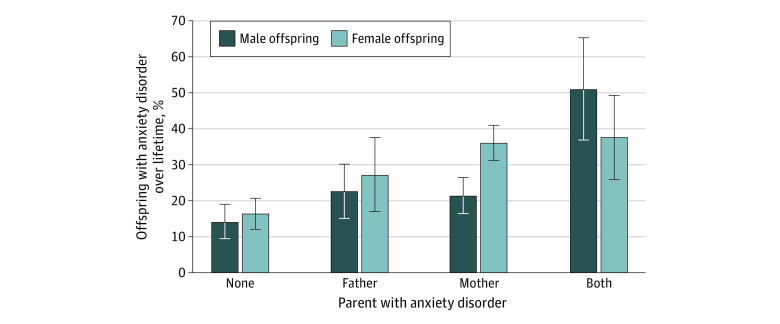
Lifetime Anxiety Disorders in Offspring by Parental Anxiety Disorder Error bars represent 1 SE.

The rates of lifetime anxiety disorders were lowest in offspring of 2 parents without an anxiety disorder (42 of 177 [23.7%]), intermediate in offspring of 1 parent with an anxiety disorder (54 of 192 [28.1%]), and highest in offspring of 2 parents with anxiety disorders (12 of 29 [41.4%]) (Figure 1). The odds of a lifetime anxiety disorder diagnosis in offspring increased proportionately to the number of parents with anxiety disorders (odds ratio [OR], 2.22; 95% CI, 1.38-3.57; *P* = .001).

### Parent Mood Disorders and Anxiety Disorders in Offspring

The rate of lifetime anxiety disorders was lowest among offspring of parents with schizophrenia (2 of 32 [6.3%]) and highest among offspring of parents with bipolar disorder (32 of 89 [36.0%]). The rates of lifetime anxiety disorders in offspring of parents with bipolar disorder were higher than in offspring of parents with no major mood or psychotic disorder (OR, 3.20; 95% CI, 1.27-8.04; *P* = .01). The rates of lifetime anxiety disorders in offspring of parents with depression were intermediate and did not differ significantly from the offspring of parents with no major mood or psychotic disorder. The odds of lifetime anxiety disorder diagnosis increased with the number of parents with mood disorders (OR, 1.75; 95% CI, 1.11-2.74; *P* = .02).

### Relative Contributions of Parent Mood and Anxiety Disorders

Because mood and anxiety disorders in parents were often comorbid and were both associated with anxiety disorders in offspring, we completed an analysis to determine the unique contributions of parent mood and anxiety disorders. When the number of parents with major mood disorders and the number of parents with anxiety disorders were both tested as independent variables in a multiple regression, parent anxiety disorders were significantly associated with increased rates of lifetime diagnoses of anxiety disorders in offspring (OR, 1.97; 95% CI, 1.16-3.36; *P* = .01), but parent mood disorders were not (OR, 1.21; 95% CI, 0.72-2.05; *P* = .48).

### Sex-Specific Transmission of Anxiety to Offspring

We examined the sex-specific transmission of anxiety from parents to offspring in a reduced sample of 299 offspring with diagnostic information on both biological parents. Anxiety disorder in a same-sex parent (OR, 2.85; 95% CI, 1.52-5.34; *P* = .001) but not in an opposite-sex parent (OR, 1.51; 95% CI, 0.81-2.81, *P* = .20) was significantly associated with a lifetime diagnosis of any anxiety disorders in offspring. A mother's anxiety disorder (OR, 3.30; 95% CI, 1.43-7.59; *P* = .005) but not a father's anxiety disorder (OR, 1.53; 95% CI, 0.60-3.94; *P* = .38) was significantly associated with consensus lifetime diagnosis of an anxiety disorder among female offspring. A father's anxiety disorder was not statistically significantly associated with consensus lifetime diagnosis of an anxiety disorder among male offspring (OR, 2.18; 95% CI, 0.89-5.33; *P* = .09).

### Anxiety in Offspring of Parents Without Anxiety

Because offspring without diagnostic information on 1 biological parent had increased rates of anxiety disorders, we examined the association between parents without anxiety in the household and anxiety disorders in offspring. With more than one-third of offspring living in single-parent households, this analysis is distinct from a reversed association of parents with anxiety disorders, examined in previous sections. These analyses were performed in the full sample of 398 offspring.

**Figure 2.**
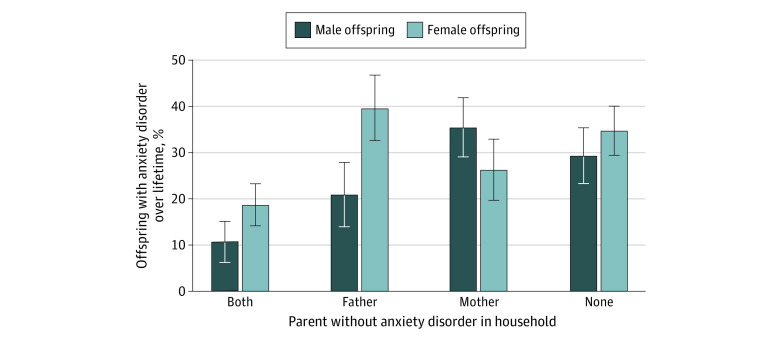
Lifetime Anxiety Disorders in Offspring by Residing With a Parent Without Anxiety Error bars represent 1 SE.

**Figure 3.**
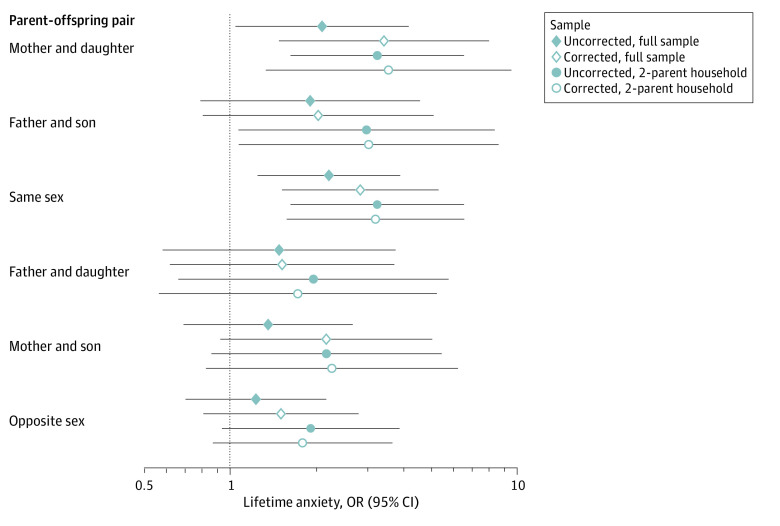
Associations of Lifetime Anxiety in Same-Sex and Opposite-Sex Parent-Offspring Pairs For each type of parent-offspring pair, we show association in the full sample (filled diamond), association corrected for the other parent’s anxiety disorder (hollow diamond), association in a reduced sample restricted to families in which both biological parents share a household with the offspring (filled circle), and the association in a reduced sample of families in which both biological parents share a household with the offspring and corrected for the other parent’s anxiety (hollow circle). Data are presented as regression estimates with horizontal lines indicating 95% CIs.

The prevalence of a lifetime diagnosis of any anxiety disorder in offspring living with 2 parents without anxiety was half (15 of 106 [14.2%]) that of offspring living with 1 (50 of 164 [30.5%]) or no (43 of 128 [33.6%]) parent without anxiety. Across the entire sample, the presence of a biological father without any anxiety disorder (OR, 0.59; 95% CI, 0.34-1.00; *P* = .05) and a biological mother without any anxiety disorder (OR, 0.64; 95% CI, 0.39-1.07; *P* = .09) in the household was not statistically associated with reduced odds of lifetime anxiety disorders in offspring. The presence of a same-sex parent without anxiety was associated with reduced odds of lifetime anxiety disorders in offspring (OR, 0.38; 95% CI, 0.22-0.67; *P* = .001), but the presence of an opposite-sex parent without anxiety was not (OR, 0.96; 95% CI, 0.56-1.63; *P* = .88). Among male offspring, the presence of a father without anxiety was associated with reduced odds of lifetime anxiety disorders (OR, 0.32; 95% CI, 0.14-0.73; *P* = .007), whereas the presence of a mother without anxiety disorder had no significant effect (OR, 0.85; 95% CI, 0.41-1.77; *P* = .67). Among female offspring, the presence of a mother without an anxiety disorder was associated with reduced odds of lifetime anxiety disorders (OR, 0.40; 95% CI, 0.19 − 0.86; *P* = .02). The presence of a father without anxiety disorder was not associated with odds of lifetime anxiety disorders (OR, 1.01; 95% CI, 0.47-2.19; *P* = .97) (Figure 2 and Figure 3).

## Discussion

In this cross-sectional study, the likelihood of offspring having an anxiety disorder increased with the number of biological parents with an anxiety disorder. This association was largely accounted for by the same-sex parent-offspring dyads. Sharing the household with a same-sex parent without anxiety, but not with an opposite-sex parent without anxiety, was associated with a decreased risk of an anxiety disorder in the offspring.

The increased risk of anxiety disorders in offspring of parents with anxiety disorders is well established.^8,9^ An increased propensity to anxiety has also been described in offspring of parents with major mood disorders.^29,30,31,32^ Based on our findings, compared with parental mood disorders, anxiety in parents appears to be more closely associated with anxiety in offspring. The risk of anxiety disorder in an individual offspring may depend on which parent is affected. We found that the transmission of anxiety disorders is largely accounted for by same-sex transmission. The sex-specific pattern is particularly pronounced when looking at parents who reside with their children; sharing the household with a parent without anxiety was associated with a lower likelihood of an anxiety disorder only when the parent was of the same sex as the offspring. This finding hints at mechanisms of the intergenerational transmission of anxiety. Although the opposite-sex transmission of psychosis is often interpreted as genetic and implicating the X chromosome,^17,18^ there is no clear genetic explanation of the same-sex transmission of anxiety disorders. Although we cannot exclude an unusual genetic mechanism contributing to the sex-specific transmission of anxiety, it is likely that learning and modeling play a significant role. This theory has been suggested before by observational and experimental studies demonstrating that children model parents’ fearful responses^20,21,33,34,35^ and that overprotective parenting increases the likelihood of anxiety in children.^36,37^ Our finding is also in line with a children-of-twins study,^16^ which concluded that transmission of anxiety from parents to children is primarily environmental. Although the current study is the first to suggest that the transmission of anxiety is at least partially specific to the same-sex parent-offspring pairs, evidence exists that other complex features, including obesity, physical activity levels, and suicidal behavior, may be more often transmitted to the offspring from the same-sex parent than from the opposite-sex parent.^38,39,40,41^

Genetic contribution to anxiety is well documented.^14,42^ The current result should not be interpreted as evidence against genetic effects. It is more likely that environmental factors, including parenting, shape this disposition into an anxiety disorder or contribute to the child’s resilience to anxiety as demonstrated by adoption studies.^43,44^ For the youth in our sample who were at a higher-than-average risk for psychopathology, the importance of parenting may have been particularly strong.

Research on parents and children has traditionally focused almost exclusively on mothers. However, the awareness of the importance of fathers for children's development has been increasing in recent years.^45,46^ Our findings suggest that sharing the household with a father without anxiety may protect children, especially sons, from anxiety.

### Clinical Implications

Anxiety disorders are treatable in children and young people across the age range.^47,48,49^ Evidence also indicates that cognitive behavioral therapies are effective for decreasing the likelihood of anxiety onset and maintenance in young children with behavioral inhibition,^49^ a well-established risk factor for anxiety disorders.^50^ Children who have behavioral inhibition as well as a same-sex parent with an anxiety disorder may have the greatest need for preventive interventions.

If modeling and vicarious learning account for a proportion of transgenerational transmission of anxiety, treating parents with anxiety disorders is important. Such treatment may be especially crucial for the same-sex offspring of parents with anxiety disorders. Treating anxiety in parents with and without mood disorders may be protective against anxiety disorders in their offspring because the intergenerational transmission of anxiety seems to be largely driven by the anxiety rather than the mood disorder in the parent.

### Strengths and Limitations

This study has some strengths. To our knowledge, this is the first report of sex-specific intergenerational transmission of anxiety disorders. The study benefits from separate assessments of the parental and offspring psychiatric disorders by raters unaware of the diagnoses in other family members and from availability of diagnostic information on both biological parents in three-quarters of the participants.

This study also has some limitations. The mean offspring age of 10 years in our sample is relatively low. Because mood disorders generally begin later in life than anxiety disorders,^2,51^ we were not able to establish the association between parental psychiatric disorders and the onset of mood disorders in children. We did not systematically collect data on current anxiety disorders in parents and hence are unable to make any conclusions about whether parental recovery from anxiety disorder decreases their children’s risk of developing an anxiety disorder. We did not control for childhood adversity or socioeconomic status. Finally, given the 2-way feedback loop between children’s and parents’ anxiety,^52^ we cannot exclude a reverse causality.

##  Conclusions

Future multigenerational studies should collect data on sex-specific transmission of anxiety disorders. Focusing on lifetime as well as current anxiety disorders in parents and offspring may elucidate whether parents’ recovery from anxiety disorders prevents the onset or maintenance of anxiety disorders in their offspring. Studies evaluating preventive interventions for children of parents with anxiety should assess whether outcomes differ in the same-sex and opposite-sex parent-child pairs.

This study’s findings suggest that the intergenerational transmission of anxiety disorders is largely accounted for by the transmission from the same-sex parent. Treating parents with anxiety disorders may protect their offspring, especially their same-sex offspring, from developing an anxiety disorder regardless of parental mood disorder.
